# NF-kB’s contribution to B cell fate decisions

**DOI:** 10.3389/fimmu.2023.1214095

**Published:** 2023-07-18

**Authors:** Caitlyn Guldenpfennig, Emma Teixeiro, Mark Daniels

**Affiliations:** ^1^ Molecular Microbiology and Immunology, University of Missouri, Columbia, MO, United States; ^2^ NextGen Precision Health, University of Missouri, Columbia, MO, United States

**Keywords:** NF-kB, B cell, B cell development and differentiation, B cell deficiency, memory, immune memory, B cell memory subsets, B cell memory

## Abstract

NF-κB signaling is essential to an effective innate and adaptive immune response. Many immune-specific functional and developmental outcomes depend in large on NF-κB. The formidable task of sorting out the mechanisms behind the regulation and outcome of NF-κB signaling remains an important area of immunology research. Here we briefly discuss the role of NF-κB in regulating cell fate decisions at various times in the path of B cell development, activation, and the generation of long-term humoral immunity.

## Introduction

**Figure 1:**
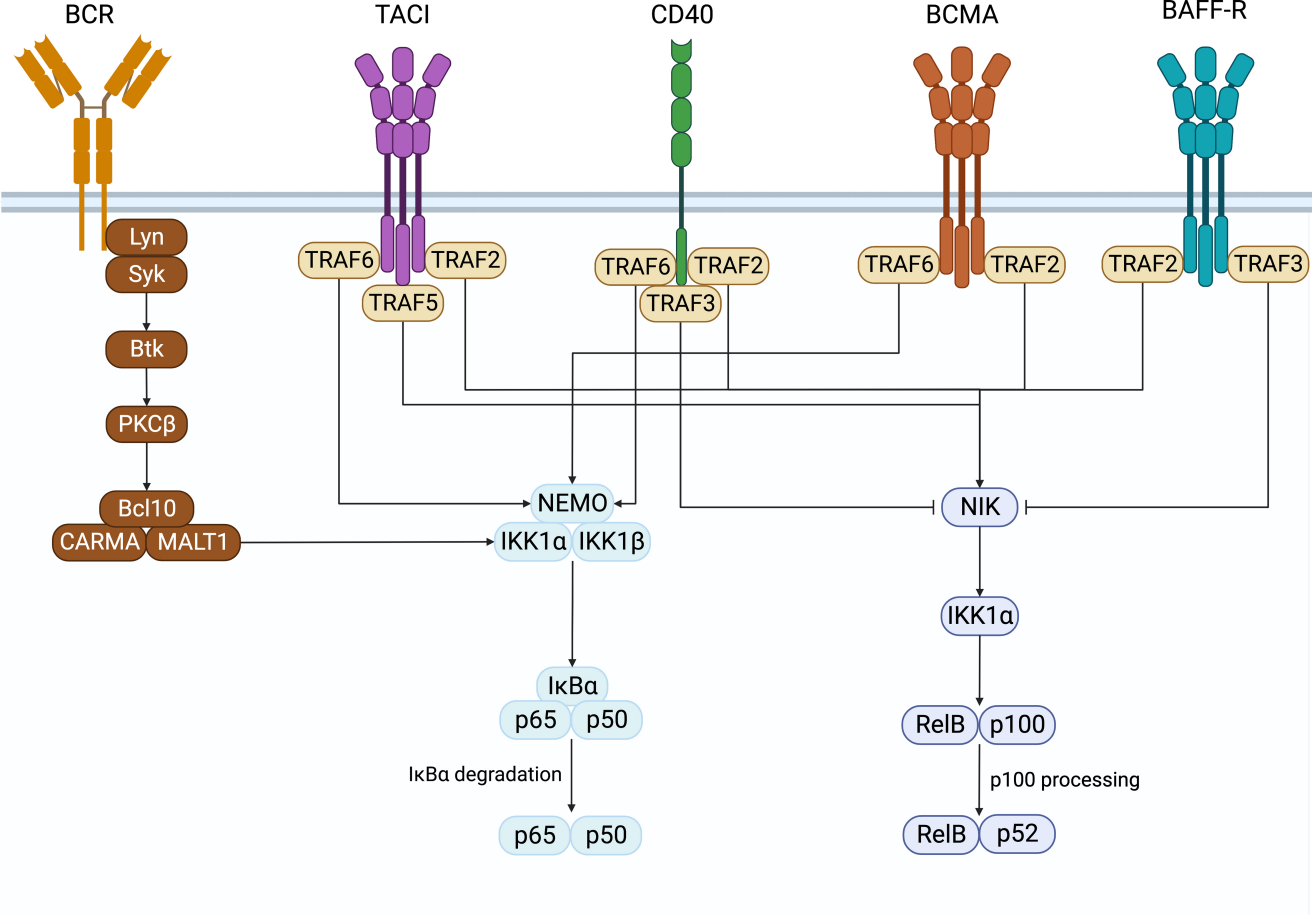
NF-κB signaling. Surface receptors expressed on B cells that induce the activation of the canonical and non-canonical NF-κB signaling pathways. Created with BioRender.com.

In B cells, NF-κB activation was first discovered in the search for transcription factors that regulate immunoglobulin gene recombination (1, 2). Classically, NF-κB is sequestered in its inactive form in the cytoplasm in a complex with inhibitory IκB proteins. There are two pathways of NFκB signaling, namely the canonical and non-canonical pathways (**Figure 1**). These contain unique members of the NFκB family members whose signals are initiated by the activation of specific receptor proximal signals that lead to the stimulation of their target NFκB complex and the translocation to the nucleus to regulate gene transcription. In B cells, the canonical pathway is activated by B cell antigen receptors (BCR), TNFa, IL-1, lipopolysaccharide (LPS), and others. It involves the phosphorylation and degradation of IκB proteins by the IκB kinases a and b (IKKa and IKKb). This releases and activates NF-κB heterodimers consisting of p50 (NFκB1) associated with REL-A (p65) or REL-B, which translocate to the nucleus and regulate the expression of target genes (3, 4). The noncanonical pathway is activated when stimuli such as B cell-activating factor (BAFF), proliferation-inducing ligand (APRIL), or CD40 ligand bind to receptors such as the BAFF receptor (BAFF-R), B cell maturation antigen (BCMA), transmembrane activator and calcium-modulating cyclophilin ligand interacting protein (TACI), or CD40, to activate IKKa. This leads to phosphorylation and proteolytic processing of the C-terminal ankyrin domain of p100 and results in the release of p52 (NFκB2)-REL-B heterodimers to activate gene expression (5, 6).

**Figure 2:**
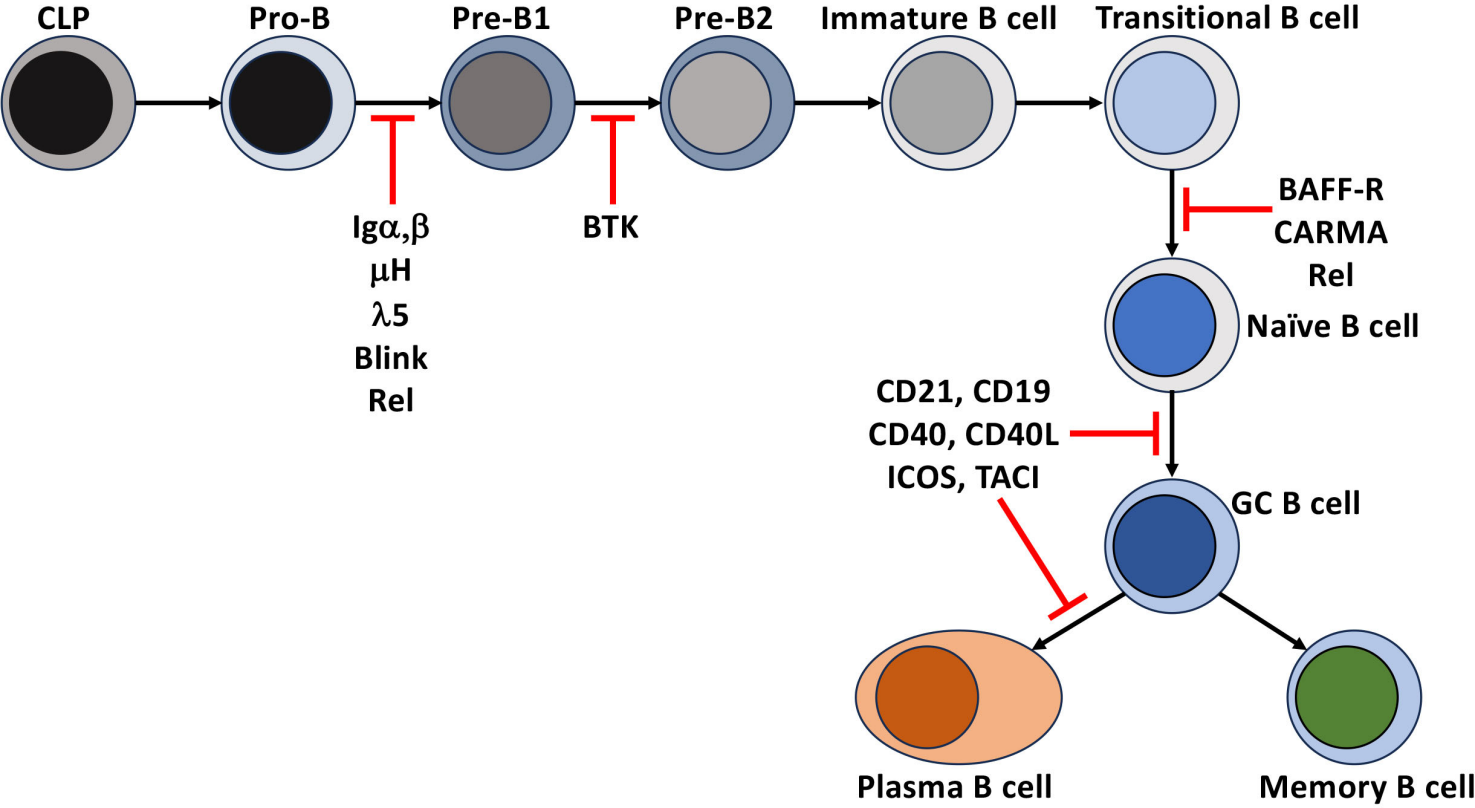
Disruption of NF-κB leads to immunodeficiencies. Points in B cell development and differentiation where mutations, polymorphisms, and deletions of receptors and intermediates of the NF-κB signaling that lead to a block in the development and differentiation of human B cells.

The genes induced and signaling pathways influenced by NF-κB play a fundamental role in B cells’ development, differentiation, and survival. It is important to understand the mechanisms that govern target-gene selection by NF-κB, the crosstalk between multiple receptors that can activate NF-κB and the crosstalk between the canonical and non-canonical pathways. NF-κB signaling can also intersect other signaling pathways to influence immune cell fate. Additionally, the outcome of NF-κB activation and the receptors that lead to its activation depends on the developmental or differentiation state and environment of the given B cell. Much of what we know about the mechanisms that regulate NF-κB signaling comes from murine models with engineered deficiencies in the signaling pathway and from human common variable immunodeficiency (CVID) patients with genetic defects and polymorphisms that disrupt NF-κB signaling (**Figure 2**). Advancing our understanding of these phenomena will aid in our ability to improve vaccine design and address health issues initiated by dysregulation of signaling and mutations in members of the NF-kB signaling pathway, including immunodeficiencies, B cell leukemia, and lymphoma, and autoimmunity, as reviewed in (7–11). This review summarizes how the different NF-κB signaling pathways and their cooperation with signals from co-receptors, B cell receptors, cytokines, inflammation, and T cell help contribute to a healthy and effective B cell compartment.

## B cell development

The development and survival of immature B cells rely largely on NF-κB signals downstream of the BCR, BAFF, and APRIL receptors. During B cell development, the pre-BCR and BCR activate the canonical NF-κB pathway to regulate central tolerance (positive and negative selection), survival, and differentiation. BAFF and APRIL are also essential for B cell development. They differentially bind to three receptors expressed on the surface of B cells. BAFF binds the B cell activating factor receptor (BAFF-R) (12), BCMA, and TACI. In contrast, APRIL binds to only BCMA and TACI. These receptors are part of the TNF receptor superfamily that can activate the non-canonical NF-κB pathway. In this section, we will briefly discuss the roles of NF-κB signaling in immature B cells’ selection, survival, and maturation.

Tonic pre-BCR and BCR signaling (signals that occur independently of receptor engagement) in developing B cells support positive selection and developmental progression through the BCR proximal signaling-based activation of a phosphoinositide 3-kinase (PI3K)/AKT and PKCβ/NF-κB. In the pro-B cell, IL-7 receptor signaling induces Rag1/2 dependent recombination of the *Igh* loci and expression of the transmembrane form of the μ-Heavy chain (μIg) and the surrogate light chain (Λ5 and VpreB) that, together with Igα, Igβ, make up the pre-BCR and, in addition to serving as a developmental biomarker, provides signals necessary for the transition to the pre-B stage of early B cell development. The contribution of NF-κB to this process was determined when the loss of signals through the pre-BCR by deletion of Igα (CD79a -/-) in pro-B cells or in models deficient in Rel induces the death of pre-B cells developing in the bone marrow. Overexpression of Bcl-2 in both models rescued the defect, indicating that the activation of NF-κB induces Blc-xL and Bcl-2 to promote their survival (13, 14). Similar blocks in B cell development were observed in human common variable immune deficiency (CVID) patients unable to express components of the pre-BCR or components of the BCR signaling pathway, BLINK or BTK. However, these defects led to more severe immunodeficiencies in humans than in mice (15–17).

Signaling by the unligated surface expression of the pre-BCR (and later in development by the BCR) activates receptor proximal signals, which in turn leads to the activation PI3K and PLCγ2/PKCβ, that activate AKT and canonical NF-κB respectively, and aid in the developmental progression of the B cell precursor (18, 19). During positive selection, AKT phosphorylation of forkhead box protein O1 (FOXO1) blocks FOXO1 nuclear localization, stopping the RAG1/2 expression. This increases MYC expression and activity, which induces several rounds of proliferation and cooperation with AKT to support survival, likely through negative regulation of the expression of the pro-apoptotic molecule Bim. This, along with the NF-κB-based induction of Bcl-xL and Bcl-2, supports the survival of developing B cells. As the proliferation of pre-B cells ends, cessation of AKT activity ends the restriction on FOXO1 activity (20). As a result, IL-7 signaling is reduced. The continued NF-κB-based induction of IRF4, along with the expression of PU.1, leads to the downregulation of the surrogate light chain and induction of the rearrangement of the *IgL* (light chain gene). The expression of IgL that successfully pairs with the previously generated μIgH results in the surface expression of the BCR (20–22). Continued induction of tonic signaling through the newly formed BCR continues to support the completion of positive selection leading to developmental progression and export from the bone marrow as newly formed transitional stage immature B cells (23–25) that continue to be dependent on tonic BCR signaling and NF-κB for their survival and next stage of development.

On the other hand, to avoid the development of autoreactive B cells, BCR ligation of self-antigens during the early stages of B cell development in the bone marrow promotes negative selection through NF-κB-induced receptor editing of the self-reactive BCR or apoptosis (19). Here the developmental stage-specific bi-phasic or bi-modal signaling of NF-κB becomes evident (22). Interestingly, increased signaling through BCR ligation activates the NF-κB signaling pathway and appears to coordinate both IRF4 and IL-7 pathways to enable the synergistic induction of light-chain recombination. The engaged BCR signals through NF-κB to increase IRF4 levels, thereby inducing light chain recombination. IRF4 targets the immunoglobulin 3’Ekappa and E-lambda enhancers to make the kappa and lambda more accessible to RAG1/2. In addition, reduced activation of IL-7 signaling activates the E-kappa promoter through E2A. IRF-4 also induces the expression of chemokine receptor CXCR4 to promote the migration of pre-B cells away from IL-7-expressing stromal cells, ensuring the reduced availability of IL-7 (20). NF-κB signaling promotes the survival of these cells through the induction of the PIM2 and increased expression of Bcl-xL and Bcl-2 *reviewed in* (19). Allelic exclusion of IgH during this process is critical to blocking the ability of B cells to express more than one BCR. Interestingly, B cells deficient in PLCγ1,2 (upstream of NF-κB) cannot execute the process of allelic exclusion (26, 27). However, whether NF-κB directly regulates this process or not is unclear (28). Here again, once a B cell that was directed to undergo receptor editing successfully expresses a new BCR that does not recognize self, the selection process continues and leads to export from the bone marrow as newly formed transitional stage B cells.

## Peripheral selection and lineage commitment

Once these immature B cells leave the bone marrow and enter the secondary lymphatics, even though the rules change slightly, they continue an NF-κB-dependent maturation and lineage commitment to become follicular or marginal zone B cells. These newly formed, immature B cells can be divided into two subsets. They enter the spleen and lymph nodes as transitional stage 1 (T1) B cells and subsequently progress to transitional stage 2 (T2) and T3. Splenic T1 B cells remain targets of negative selection. Unlike their bone marrow precursors, BCR engagement of self leads to apoptosis (29). This ensures that B cells expressing BCRs that recognize self-antigens, either not present in the bone marrow or that are only present in the periphery, are deleted before they become competent to express effector functions. This is supported by studies in mice (29) and humans (30–34) that demonstrated the negative selection of high-affinity self-reactive BCR, and a subsequent narrowing of BCR repertoire diversity in naïve B cell populations when compared to their transitional B cell counterparts. T1 B cells require activation of NF-κB to survive and mature to the T2 stage of development. This was demonstrated by showing that deletion of Rel and RelA blocks peripheral B cell development at the T1 stage (35). The Rel deficient T1 B cells are susceptible to death due to defects in the expression of survival factors Bcl-2 and A1 (36). Human patients with a homozygous deletion of CARMA (also known as CARD11) (**Figures 1**, **2**) abrogated BCR-based activation of the canonical NF-κB pathway while CD40 signaling remained intact. Additionally, BAFF-R expression was also inhibited in these cells, and they were blocked at the transitional stage of development (37). Importantly, BAFF signaling through BAFF-R is required to induce the maturation from T1 to T2. The engagement of BAFF-R activates the non-canonical NF-κB signaling pathway and is essential for this process. In BAFF-deficient mice, like the Rel-deficient animals, all mature B cells are also lost after the T1 stage of maturation (38). This was also seen in BAFF-R signaling mutants and BAFF-R deficient mice and humans (39–41). The loss of BAFF-dependent NF-κB signaling resulted in a decrease in the levels of Bcl-2/Bcl-xL (42) and increased apoptosis of B cells in the periphery. Constitutive expression of Bcl-2 overcame this phenotype to suggest that the loss of mature B cells was due to a loss of non-canonical NF-κB signaling through the BAFF-R (38, 39, 42, 43). These findings were extended and determined that canonical NF-κB signaling was also induced by BAFF early. Thus, the upregulation of Bcl-xL promoted the survival of T1 B cells until BAFF-R-dependent non-canonical (NF-κB2) signaling could take over to promote their maturation and survival (40, 44). While the survival signal provided by BAFF through BAFF-R was thought to be required for all mature B cells, loss of IKKα activity downstream of NIK surprisingly had no impact on mature B cell survival if this activity was lost after maturation had occurred (45). In fact, naïve human follicular and marginal zone B cells are highly dependent on BAFF for survival as a genetic deficiency or pharmacologic inhibition of BAFF depletes these naïve B cell pools. In contrast, memory B cells (MBC) are less dependent on BAFF for survival as in patients treated with the therapeutic anti-BAFF monoclonal antibody, belimumab, the MBC pool appears unaffected (46, 47). This implies that fully mature resting B cells may not rely heavily on BAFF-R-mediated NF-κB signals through the NIK-IKKα axis but may still depend on canonical NF-κB and other signaling pathways downstream of the BAFF-R to survive in the periphery. Thus, BCR-dependent canonical and non-canonical NF-κB activity through BAFF are both necessary for generating a complete and functional repertoire of peripheral mature naïve B cells.

The BAFF-R/BCR axis also controls the lineage commitment to the follicular or marginal zone B cell fate (48). The most abundant population of mature B cells in mice and humans are follicular B cells. These are recirculating cells that home mainly to B cell follicles and thus are well suited to perform their function in T cell-dependent immune responses (48). Marginal zone B cells (MZB) are innate-like antibody producers located at the interface between the white pulp of the spleen and the circulation and rapidly respond to blood-borne antigens. They have been reported to be able to self-renew and survive for very long periods of time (49). Determining the follicular versus marginal zone B cell fate depends on integrating NF-κB signals generated by the BCR and BAFF-R and crosstalk/integration of signals from Notch2 (8, 48, 50). MZBs develop in mice and humans in the presence of weak BCR signals, canonical NF-κB, and Notch2 (8, 48, 51, 52). As mentioned above, BAFF-R deficiency leads to a total loss of mature B cells. Interestingly, the expression of constitutively active IKKβ (a component of the canonical NF-kB pathway) can rescue the MZB development (53, 54). Since this does not occur in BAFF-R/CD19 double knockout mice, this indicates there is cooperativity between AKT and NF-κB in the commitment to the MZB lineage (48–52).

Follicular type II B cell lineage fate occurs in the presence of tonic B cell receptor signaling, mediated by Igα/Igβ (55), and BAFF-R signals, including non-canonical NF-κB. Importantly, tonic BCR signaling increases the expression of p100 as a crucial signaling intermediate downstream of BAFF-R, providing a mechanism for this cell fate (56). On the other hand, the follicular type I B cell lineage arises from strong BCR signals and canonical NF-κB. Here PI3K and BTK function downstream of the BCR to facilitate the activation of canonical NF-κB, necessary for the survival of developing follicular type I B cells (57–59). Despite the wealth of data on the BCR/BAFF developmental axis, the lack of clarity in some experimental models and how the balance between the signals from BCR, BAFF-R, CD19, BAFF levels, or other receptors leads to cell fate outcome requires a more detailed examination of the process.

## Naïve B cell activation

In an immune response, follicular B cells, primed by their cognate antigen and TLR-based inflammatory cues, become activated and traffic to the T cell zone, where they interact with CD4 + follicular helper T cells (T_FH_) (60). Newly activated B cells receive help from T cells expressing CD40L and cytokines and, in conjunction with antigen-dependent BCR signals, inflammation, and TLR (61), some differentiate into short-lived antibody-secreting cells in the follicles or pre-germinal center (GC)/GC-independent memory B cells. B cells that do not receive help from T_FH_ die. Others acquire the gene and protein expression profile that enables their entry into the germinal center (62–64).

## Germinal center reactions

Within a week of antigen exposure, GCs develop in the center of B cell follicles in the spleen and lymph node (65). As the GC matures, it polarizes into a dark zone (DZ), proximal to the T cell zone, and a light zone (LZ), proximal to the lymph node capsule or the marginal zone of the spleen, extensively reviewed in (66, 67). The DZ consists almost entirely of GC B cells and a network of reticular cells and appears “dark” by light microscopy. By contrast, the LZ, which also gets its name from its “lighter” appearance, contains a network of follicular dendritic cells (FDC), CD4+ T_FH,_ and GC B cells. The DZ functions as the location where selected GC B cells proliferate and undergo somatic hypermutation (SHM) with the goal of increasing BCR affinity for their cognate antigen. After SHM, the GC B cells are directed to traffic to the LZ where their newly modified BCR are selected on antigen bound to FDC and interactions with their cognate T_FH_. The positive selection signals from this interaction lead to the induction of proliferation and return to the DZ to start the process over. The selection of GC B cells, the cycling between the DZ and LZ, and the decision to leave the GC as memory B cells or long-lived plasma cells require or are significantly influenced by NF-κB (62, 66–69) and are discussed briefly below.

Murine and human GC-B cells receive help from T_FH_ in the form of cytokines (IL-4, IL-21, IL-10), BAFF, APRIL, and CD40L that, together, enable B cells to activate their class-switching machinery and undertake the process of somatic hypermutation required for developing BCR with higher affinity for antigen and high-affinity memory B cells or long-lived high-affinity antibody-producing plasma cells (PC) (63, 70–73). CSR and SHM require the expression of activation-induced cytidine deaminase (AID). DNA methylation and gene expression data from human tonsillar GC B cells, identified genes of the NF-κB signaling pathways that were de-methylated and highly expressed, including those targeted by AID (74). The CSR machinery that controls specific antibody classes is induced by transcription factors downstream of specific inflammatory cytokine receptor signals. For example, on activated B cells, signaling through the IFNγ receptor activates STAT1 to induce T-bet and promote class switching to IgG2a. Similarly, TGFβ signaling through SMAD and RUNX promotes class switching to IgA and so on (75–77). BAFF and APRIL contribute to the CSR due to TACI-induced activation of the canonical NF-κB pathway through MyD88 to induce AID expression (78, 79). Furthermore, the transcriptional regulator BATF, downstream of BCR and CD40-induced NF-kB (80), is also required in B cells to generate germline switch transcripts and to promote AID expression.

BAFF-R is important for maintaining the GC reaction (18). However, the BAFF-R-dependent mechanism and signaling pathways required for this maintenance of the GC have not been fully resolved. B cell-intrinsic non-canonical NF-κB signaling is required for GC formation (49), which may be initiated through the BAFF-R. Still, while BAFF and APRIL are common ligands that induce non-canonical signaling, the induction of NF-κB2 could also be coming from other factors/receptors. TACI has been demonstrated to negatively regulate the germinal center reaction in part by activating cIAP to ubiquitinate NIK for degradation leading to the inhibition of BAFF-R mediated non-canonical NF-κB survival in the germinal center (81, 82). TACI also induces BLIMP-1 expression to inhibit the GC reaction and drive the generation of long-lived plasma cells. Thus, the canonical and non-canonical NF-κB pathways through BAFF-R and TACI are important for regulating the B cell antibody response.

BAFF and APRIL also have key roles in memory B cells and long-lived plasma cell generation and survival. Early studies showed that BAFF and APRIL were not required for memory B cell survival (83, 84). In contrast, more recently, it has been shown that memory B cells need BAFF and BAFF-R to survive in the periphery in mice and in a subset of human memory B cells (85–87). Additionally, Muller-Winkler et al. determined that the NF-κB signals downstream of the BAFF-R are differentially necessary for memory B cells (87). IgM+ memory B cells required both IKKα and IKKβ for optimal survival, while IgG+ memory B cells only relied on IKKβ. Long-lived plasma cells also need BAFF/APRIL signaling to survive, and the signals are thought to occur primarily through BCMA in mice and humans (83, 88, 89). This may be due to IKKα specific signaling, as loss of the signaling through the NIK-IKKα axis led to decreased long-lived plasma cells (90). BCMA also drives memory B cell differentiation toward the plasma cell fate (89, 91, 92). Although, whether this is directly through NF-κB or through the upregulation of BLIMP-1 remains to be determined. BAFF-R deficient CVID patients have severely reduced serum IgM and IgG levels and impaired T cell-independent responses against pneumococcal polysaccharides. However, like BAFF deficient mice, IgA levels and gut IgA^+^ plasma cell numbers in these patients were normal, *reviewed in* (16, 89). Deletion of TACI or APRIL in mice and human patients expressing mutant TACI have significantly reduced IgA levels (93–96). Low IgA and IgG antibody titers in TACI-deficient patients suggest its importance for CSR in human B cells. It has also been suggested that decreased numbers of BAFF-binding receptors caused by the absence of TACI give rise to more circulating BAFF binding to BAFF-R, thus supporting B-cell survival (97). High levels of BAFF in mice and humans increase the numbers of mature B cells and may be the mechanism that leads to progressive autoimmune disease in these B cell-deficient patients (89, 98).

## Help from CD40 et al.

CD40 is a costimulatory molecule expressed on B cells and other antigen-presenting cells and contributes to B cell proliferation, antibody class-switching, and rescues B cells from activation-induced apoptosis (99–102). CD40 has been shown to be capable of activating both the canonical and non-canonical NF-κB pathways by interacting with TRAFs (103–105). TRAF2, TRAF3, and TRAF5 have all been shown to associate with CD40 and are important for activating the CD40-induced non-canonical NF-κB signaling (105). CD40’s interaction with either TRAF2 or TRAF3 is also important for inducing canonical NF-κB signaling, while interacting with both is required to induce non-canonical NF-κB (100). Additionally, TRAF6 associates with CD40 to initiate the canonical NF-κB signaling pathway (105).

Like BAFF-R, CD40 plays an important role in B cell antibody class switching and is able to induce class switching alone or in combination with cytokines and the BCR (99). CD40’s ability to activate NF-κB signaling is essential to this process. Jabara et al. demonstrated that mice with B cells expressing CD40 proteins unable to interact with TRAF2 or TRAF3 alone had decreased serum IgG and IgE and smaller germinal center reactions in response to T cell-dependent antigens (100). Furthermore, B cells expressing CD40, unable to bind to both TRAF2 and TRAF3, had further reduced serum IgG and IgE and undetectable germinal center reactions. Ultimately, B cells expressing a mutant form of CD40 that could not signal through TRAF2 and TRAF3 could still induce the canonical NF-κB pathway but not the non-canonical NF-κB pathway. In contrast, CD40 double mutant proteins could not induce either (100). These data demonstrated that CD40’s ability to interact with both TRAF2 and TRAF3 and induction of both the canonical and non-canonical NF-κB signaling is important for CD40-mediated antibody class switching. A second study also demonstrated the reliance of CD40 on canonical NF-κB to induce class switching by inducing AID expression (102). This was shown when B cells deficient in p50 or c-Rel could not induce AID expression in response to CD40, while by contrast, p52 deficient cells could, although to a lesser extent than the wild-type B cells. Thus, both the canonical and non-canonical NF-κB pathways downstream of CD40 are required for CD40-mediated AID expression and antibody class switching.

CD40-mediated NF-κB signaling also regulates other aspects of the B cell response, including the germinal center reaction, B cell trafficking, and memory B cell differentiation. The NF-κB p50/p65 heterodimer binds to the IRF4 promoter and induces its expression downstream of CD40 activation. IRF4 expression can negatively regulate the germinal center reaction by binding to the Bcl-6 promotor and downregulating its expression (106). Thus, CD40 can negatively regulate the germinal center reaction and promote B cell exit from the germinal center reaction as post-GC memory B cells. Additionally, CD40-induced non-canonical NF-κB signaling is important for CXCR5 expression (107), which helps localize newly activated B cells to the follicles to traffic toward and receive help from T_FH_ cells (108). CXCR5 and CXCR4, dependent at least in part on NF-κB signaling, participate in the cycling of GC B cells between the LZ and DZ reviewed in (62, 64, 67). Interestingly, GC in patients with genetic defects in CD40 are poorly organized or nonexistent (109, 110). Finally, CD40 and its induction of NF-κB signaling regulate the differentiation of CD80+PD-L2+ vs. CD80-PD-L2- and CD80-PD-L2+ memory B cells. These two subsets have been shown to be functionally distinct, where CD80+PD-L2+ memory B cells primarily differentiate into plasma cells upon restimulation, whereas CD80-PD-L2- memory B cells primarily generate new germinal center reactions (111). Interestingly, strong CD40 leads to higher expression of CD80 by inducing IRF4 and BAFT expression through NF-κB signaling, which then forms a heterodimer able to bind to the CD80 promotor and induce its expression (80). Thus, CD40-mediated NF-κB signaling is essential for inducing and regulating efficient B cell primary and secondary B cell responses.

## GC selection decisions

Multiple mathematical and experimental models that analyze the LZ to DC cycle indicate that 10%–30% of B cells that traffic into the LZ are selected to re-enter the DZ. The remainder either die by apoptosis or exit the GC as memory B cells or PC (67, 68, 112). T cell help drives cycle reentry and GC selection. In addition, the nature of the B cell-T_FH_ cell interactions regulates the number of divisions and the cell cycle speed of selected B cells in a manner proportional to the strength of that interaction (69, 113, 114). Here we will discuss the contribution of NF-κB signaling to this process.

Recruitment and retention of B cells in the GC require them to upregulate the zinc finger transcription factor B cell lymphoma 6 (BCL-6). This is mediated by antigen-dependent BCR signals and engagement of CD40 by T_FH_ expressing CD40L and cytokine (115, 116). T_FH_ cell expression of IL-4 and IL-21 acts directly on B cells to promote the expression of BCL-6 (117, 118). BCL-6 is a transcriptional repressor that blocks the upregulation of migratory cell receptors S1PR1 and Gpr183. BCL-6 also upregulates the expression of S1PR2, which acts as a retention signal to maintain B cells in the GC (116). BCL-6 regulates the expression of a vast network of genes controlling cellular processes, including the DNA damage response, apoptosis, BCR, and CD40 signaling, inhibition of plasma cell differentiation, and T cell/B cell interactions (62, 64). NF-κB dependent induction of IRF4 is necessary for the initiation, but not the maintenance, of the GC response. Interestingly, transient expression of IRF4 in B cells induces the expression of BCL-6, AID, and POU2AF1 and promotes GC development (119). IRF4 expression is rapidly induced by BCR stimulation and CD40 through the activation of NF-κB (36). However, sustained IRF4 expression is sufficient to directly repress BCL-6 expression and promote plasma cell differentiation, which may reflect the kinetic and dose-dependent function of IRF4 in GC-B cells (36, 37). In-depth genetic analyses indicated that IRF4/PU.1 or BATF bound to Ets or AP-1 motifs to control transcription factors PAX5, BCL-6, and BACH2, which contribute to retention in the GC through negative regulation PC differentiation (120). Conversely, higher concentrations and sustained expression of IRF4 promoted the generation of plasma cells while antagonizing the GC fate (119). The sustained expression of IRF4 upregulates BLIMP1 and downstream target XBP1, transcription factors that direct GC B cells to become long-lived plasma cells (121). Mechanistically, higher concentrations of IRF4 shifted binding to genetic interferon response sequences to induce genes that inhibit the GC program. Expression of Blimp1 terminates the expression of BCL-6 and BACH2, responsible for the maintenance of the GC phenotype, to drive cells to become PC (120, 122–125). Conditional deletion of IRF-4 in GC B cells blocked the generation of post-GC plasma cells, as IRF-4 deficient memory B cells were unable to upregulate BLIMP1 and to differentiate into plasma cells (121). The NF-kB subunits c-Rel and RelA had different roles in this process. Signaling through c-Rel was critical for maintaining newly formed GC, while RelA was dispensable for this process. Importantly, RelA was able to directly induce BLIMP1 independently of IRF-4 and is, therefore, essential for PC development (126). Together, these data indicate that the NF-kB signaling is important for determining the GC and PC fate through the regulation of the IRF-4/Bcl-6/BLIMP1 axis (120, 125, 127). Understanding what signals induce the activation of RelA *in vivo* may provide valuable clues to the cellular processes involved in PC differentiation.

To achieve a repertoire of B cells with a higher affinity to antigen, one would expect there to be a pronounced difference in how naïve and GC B cells sense or respond to antigens. One difference maps to the type of synapse GC B cells form when engaging antigen (128, 129). Naïve B cells form a classical ‘bullseye’ synapse, directing antigen internalization to a central synaptic structure. By contrast, GC B cells form small foci of synapses and internalized antigens in peripheral synapses (129). Additionally, low-affinity antigens triggered continuous engagement and disengagement of membrane-bound antigens, whereas high-affinity antigens induced stable synapse formation. The mechanical forces needed to pull antigens from the surface of FDC also provide a way to sense antigen affinity. Cytoskeletal contractility disrupted low-affinity and individual BCR-antigen interactions and promoted the internalization of high-affinity, multivalent BCR (130). Here then, these interactions increase the activation of BCR signals, likely increasing the dose of antigen processed and presented to T_FH,_ providing an indirect mechanism by which T_FH_ can sense antigen affinity. These data pose potential mechanical and biochemical mechanisms for differentiating high-affinity BCR.

Considering the NF-κB signals are activated by crosstalk between GC B cells, environmental cues and T_FH_ connects NF-κB to GC selection decisions. A consequence of the interactions between GC B cells and T cells important for the selection decision is the induction of feed-forward or positive feedback loops in both cells. ICOSL on B cells promotes upregulation of CD40L on T cells, which in turn further upregulates more ICOSL expression on the B cell. This interaction promotes the GC B cell T_FH_ interactions described as ‘‘entanglement’’ to promote the differentiation into high-affinity plasma cells (131). ICOS-deficient CVID patients lack isotype-switched B cells and there is a paucity of GC in spleens and lymph nodes (132). Using intravital microscopy, the duration of GC B cell interactions with T_FH_ was also greatly increased by specific high-affinity antigen delivery to GC B cells (113, 114). ICAM has also been implicated in these increased cell-cell interactions. This also increased the T cell production of B cell helper cytokines IL-4 and IL-21. In addition, T cells expressed more BAFF and increased the survival of B cells that have acquired high-affinity mutations (133).

Another change between naïve and GC B cells in the response to an antigen is a qualitative rewiring and quantitative dampening of BCR signaling in GC B cells (128, 129). Although antigen stimulation of BCR can trigger proximal signaling in GC B cells, overall NF-kB signaling is reduced. A critical step of BCR-based NF-kB activation appears to require additional input from T_FH_ cells (129). In fact, BCR signals in mice and humans are dampened 100-fold in GC B cells, and CD40 signaling is rewired in these cells (63, 128, 134). Using a combination of genetic ablation, specific inhibitors, and *in vivo* delivery, GC stimuli on *ex vivo* GC B cells and GC B cells *in vivo* found that CD40 induced signals *via* NF-kB but not PI3K. At the same time, BCR signals do not activate NF-kB, while BCR-dependent activation of AKT remained intact. In GC B cells, both CD40 and BCR signals are required to induce c-Myc, which is essential for their selection, survival, and proliferation in the GC. This is markedly different from naïve B cells in which either receptor can signal through both PI3K/AKT/mTOR (135) and NF-kB and to c-Myc. The phosphatase PTEN has been implicated as part of the mechanism responsible for the dampening of BCR signaling in GC B cells (136). More recent work using inhibitors of AKT indicated that differential activation of AKT by GC B cell-specific expression of PDK1 and PTEN led to enhanced negative regulation of BCR signaling through CSK, SHP-1, and HPK1 (136). Altogether, these highlight the essential role T_FH_ play in the activation of NF-kB to enhance the successful selection of high-affinity B cells in the GC reaction (67–69, 113). When considered together, these results, along with computational modeling, support a kinetic control model of the PC vs. GC B cell fate determination (62, 63, 123). However, the precise thresholds, cross talk, and synergy between inputs such as BCR affinity, antigen dose, the magnitude and nature of T cell help, and contribution from FDC remain undefined. Furthermore, quantifying these on a per B cell basis under physiological conditions *in vivo* has not yet been worked out.

## B cell memory diversity and tissue resident B cell memory

The purpose of B cell memory is to provide enhanced antibody responses and immune protection against repeat exposure to homologous or heterologous infection. The B cell memory compartment is comprised of highly diverse subsets of B cells with unique trafficking patterns, longevity, cell surface phenotype, and functions (63, 137). The signals that drive B cell memory vs. the generation of long-lived plasma cells are not worked out and are highly controversial at best. The ability of B cells to differentiate into memory B cells versus PCs changes over the course of an immune response. Memory B cell generation is favored in the pre-GC and early GC periods, and long-lived PC differentiation becomes more pronounced later in the immune response in mice and humans (138–140). Some data from these experiments suggest that lower affinity BCR favors the generation of memory over PC, especially early in the immune response. In addition, a single B cell can differentiate into each of the diverse memory subsets depending on several factors (139, 140). Considering the role of NF-kB in the regulation of the IRF-4/Bcl-6/BLIMP1 axis (120, 125, 127), it is likely to be at least part of the determination of these decisions. TLR signals provides a significant contribution to NF-kB signaling. Thus, as inflammation fades over time, the selection of GC B cells into the PC fate is more likely to be dependent on signals through the BCR, CD40, T_FH_, cytokines, and BAFF. Importantly, IL-21 has an essential role in conjunction with those signals in determining the nature of the human B cell immune response and programming of B cell memory (72, 85). Other inflammatory and environmental cues also lead to differences in memory subtypes such as the connection between inflammation and CSR INFg and IgG2 or TGFb and IgA or IL-21 and INFg, as discussed earlier (75–77). Another example when human B cells with BCR that bind TLR-7 or 9 ligands, signals from BAFF and concomitant IL-21 or IFNγ promotes T-bet^+^ B cell development (141–143).

Because the affinity of GC B cells for antigen also increases over time and BCR signaling is rewired (63, 128, 134), the temporal and antigen affinity models may encompass developmental and environmental changes along the course of the immune response that define new thresholds of signals that promote the long-lived PC vs memory B cell fate. Precisely defining this moving target *in vivo* is a difficult task. Despite this, recent studies provided additional mechanistic detail concerning the selection of GC B cells into the plasma cell lineage, showing that plasma cell lineage commitment was favored by BCL-6^low^CD69^hi^ GC B cells that expressed the transcription factor IRF4. The generation of BCL-6^low^CD69^hi^ GC B cells, which also expressed high levels of ICAM1 and SLAM, was regulated by T cell-mediated signaling through CD40, suggesting that T_FH_ cell–GC B cell interactions were key to the generation of GC B cells that were prone to differentiate into long-lived plasma cells (144).

Tissue-resident memory B cells in humans and mice have been found in non-lymphoid tissues that are phenotypically and functionally distinct from their classical lymphoid-associated counterparts (145–147). Tissue-resident memory B cells (BRM) perform protective functions in peripheral tissues dependent on their ability to engage antigen at the site of infection, secrete cytokines, and differentiate into plasma cells (147). The tissues BRM are directed to enter depend in part on the pathogen and site of infection. Much less is known about the mechanism of their generation and maintenance (147). A role for NF-κB in regulating this process is suggested by a recent model of influenza infection. Here CD40L blockade through the first 2 weeks blocked HA and NP-specific B cell response in both the LNs and the lung. CD40 blockade between day 10 and day 20 or between day 20 and day 30 allowed BRM to appear in the lung. Importantly, when CD40L was blocked between days 30 and 40, all GC B cells were lost, while flu-specific BRM cells in the lung were maintained. Since BRM generation also depended on the presence of their cognate antigen in the lung, it suggests that cooperation between CD40 and BCR (NF-kB/AKT axis)? regulates their generation. Interestingly this parallels our recent work in T cells, where temporal regulation of NF-kB controls both the generation and development of tissue-resident T cells (148).

MZ B cells are innate-like B cells specialized to mount rapid T-independent, but also T-dependent responses against blood-borne pathogens and are a major source of IgM antibodies in humans (49). Whilst there are similarities to mouse and human MZB development (52), their phenotype as memory/PC are different. Human MZB cells express hypermutated IgM and are circulatory, while their murine counterparts express unmutated IgM and remain in the spleen (149, 150). Interestingly, the MZ B cells in children less than 2 years of age express low levels of AID, while AID was not detected in MZ B cells from adults (151–153). In fact, children show a higher clonal diversity in the MZ subset than in class-switched B cells (149). Furthermore, MZB with mutated IgM found in patients with genetic defects in CD40* *suggest they can arise independent of a GC reaction (109, 110). Whether this is due to the differences in the physiology of mouse and splenic marginal zones, environmental cues, NF-κB activity, or other factors is not clear.

## Looking ahead

Our reductionist methodology and ability to design well-controlled experimental approaches have provided us with a solid picture of the function of NF-κB in humoral immunity. Other mechanistic information has come from exhaustive studies of CVID patients with various genetic deletions, mutations, and polymorphisms in NF-κB. These works have been used to develop FDA-approved treatments for inflammatory diseases and multiple cancers (154–158). However, the precise quantification of the dose of antigen or cytokine a B cell is exposed to, how many and which cell-cell encounters occur within a given time, or what is the variability in paths a cell can follow to arrive at a given cell fate *in vivo* is not able to be determined by these methods. Thus, some of the fine details in human and mouse B cell development and differentiation remain unknown. The progressive development of new ‘multi-omics’ approaches enables the potential to provide these data.

Phenotype tracking human and mouse B cells through development and differentiation has provided a consistent and high-definition classification of B cell subsets during B cell development and differentiation into MBC and PC. In addition, they have corroborated checkpoints in development in mice with those across a population of healthy human donors. RNA velocity analyses have begun to precisely map out the quantity, quality and kinetics of gene expression over the course of early development and differentiation, defining pathways of development. Lineage tracing and single-cell analyses across a diverse population have been used for the discovery of new B cell subsets and their plasticity. In addition, aligning surface phenotypes with genetic and signaling profiles provide starting points to extend our understanding of NF-κB signaling in B cell *extensively reviewed in* (159–164). These new methodologies can lead to our ability to fill gaps in our understanding of NF-κB. For example, how can we improve vaccine response using our understanding of the interplay between inflammation and long-term immunity to improve vaccine design? Is a given B cell memory subset better at providing immune protection against a pathogen? When is the best time in an immune response to manipulate NF-κB activity to direct tissue-resident B cells to (or away from) an organ of interest such as the lung to increase protection and safety of vaccines against respiratory infections?

## Oncogenesis

When working in harmony, the pathways regulating NF-κB described above lead to the generation and maintenance of a complete repertoire of the diverse subsets of healthy B cells. However, many of these pathways are susceptible to dysregulation, leading to aberrant NF-κB signaling and lymphoid tumorigenesis. Many B cell leukemias and lymphomas, including Hodgkin lymphoma, activated B-cell-like diffuse large B-cell lymphoma (ABC-DLBCL), lymphomas of the mucosa-associated lymphoid tissue (MALT), and chronic lymphocytic leukemia (CLL) are associated with constitutively active NF-κB signaling which contributes to these cancer’s proliferation and survival (165–168). While we will not cover the role of NF-κB in B cell malignancies in depth (comprehensively reviewed in (169, 170)), we will summarize a few key points at which NF-κB may be dysregulated to drive B cell transformation and other tumors.

In healthy B cells, MyD88 can coordinate the activation of B cells downstream of TLRs and induce AID expression by activating the NF-κB pathway, as described earlier. However, mutations in MyD88, most notably the L265P mutation, lead to the constitutive activation of NF-κB and are commonly found in ABC-DLCBL and can also be found in MALT lymphomas and CLL (171–174). This mutation allows poly-ubiquitination by an E3 ubiquitin ligase RNF138 which leads to activation of MyD88^L265P^ and drives activation of the NF-κB and B cells transformation (175). Ubiquitination of MyD88^L265P^ is negatively regulated by the ubiquitin editing protein A20-mediated downregulation of RNF138 and, as such, knock-down of A20 in this model enhanced the efficiency of transformation. These suggest two complementary mechanisms by which the dysregulation of NF-κB signaling is caused by MyD88 mutations. A20 down-regulates canonical NF-κB signaling mediated by multiple receptors through multiple mechanisms (176, 177). Interestingly, inactivation or deletion of A20 is also commonly linked to B cell malignancies on its own, including ABC-DLBL.

Signals from both the pre-BCR and BCR can activate NF-κB. Signals downstream of the pre-BCR and BCR signaling are commonly dysregulated in B cell malignancies, where enhanced activation or disrupted negative regulation of components of the BCR pathway leads to the constitutive activation of NF-κB (178). The chronic activation of the BCR by either self or non-self-antigens has been shown to lead to the development of lymphoid cancers (179, 180). Mutations within the CD79B and CD79A co-receptors are common and maintain this activation (181). Mutation of CD79B can increase the surface expression of the BCR and limit its internalization to maintain chronic signaling (182). As described above, engagement of the BCR leads to the activation of BTK, which in turn activates canonical NF-κB signaling. Cancers that are dependent on mutations in CD79 are sensitive to Bruton tyrosine kinase (BTK) inhibitors and, thus, downstream inhibition of NF-κB (183). A20 down-regulates canonical NF-κB signaling mediated by multiple receptors through multiple mechanisms (176, 177). Interestingly, the inactivation or deletion of A20 enhances the transformation of these cells (178, 184, 185).

Finally, the mutation of proteins within the CARMA/MALT1/BCL10 (CBM) complex also leads to constitutive NF-κB signaling and oncogenesis. In healthy B cells, the CBM complex is activated downstream of the BCR through the phosphorylation of CARMA. This phosphorylation induces a conformational change which then allows it to associate with BCL10 and MALT1. The active CBM complex will then recruit the IKK and TAK1, which will then be able to phosphorylate IKKβ and induce NF-κB signaling. Gain of function mutations within any of the three proteins that make up the CBM complex can result in the constitutive activation of NF-κB and drive B cell oncogenesis. Again, multiple leukemias and lymphomas depend on the crosstalk between AKT and NF-κB (178, 184, 186). This suggests the potential inclusion of other scaffolds, such as POSH (plenty of SH3 domains), that are implicated in crosstalk between these and other signaling pathways important to the survival of other cancers and for lymphocyte survival and differentiation (187–191).

Stepping briefly outside of the hematopoietic system, considering the general function and the genes targeted by the NF-κB signaling pathway, it has the potential to play a role in multiple tumor types. Mutations in NF-κB genes are rare in solid tumors. However, activation and nuclear translocation of RelA are found in many tumors and associated with tumor progression. In these tumors, aberrant activation of signaling molecules upstream of NF-κB, such as mutant or oncogene-driven RAS, EGFR, PGF, and HER2, likely induce increased NF-kB signaling, *reviewed in* (192–194). NF-kB then, in turn, upregulates genes that control cell survival, proliferation, metastasis, angiogenesis, and others. For example, BCR-ABL signaling in AML, CML, and B-ALL activates NF-κB, and tumorigenesis driven by BCR-ABL-expressing cells was blocked upon inhibition of NF-κB (195). In addition, NF-kB’s ability to induce the expression of AID and APOBEC was demonstrated as a mechanism for increased mutagenesis in cervical cancer (196). High levels of c-Rel have been found in non-small cell lung carcinoma (197), breast cancer (198), and squamous cell carcinomas of the head and neck (199). Defective IkBα was found in several solid tumors such as breast, colon, ovarian, pancreatic, bladder, prostate carcinomas, and melanoma (193). Taken together, the data from solid tumors and hematological malignancies provide the information that can be used to develop was to inhibit NF-κB to enhance current tumor treatment protocols.

## Conclusions

NF-κB signaling plays a major role in the development of a healthy B cell immune response. It has unique functions depending on the developmental/differentiation state of the cell and cooperation with multiple other signaling pathways responsible for cell fate decisions. In addition, the timing and kinetics of the induction of NF-κB signaling (transient vs. sustained) greatly influence the outcome of its activation. The cooperation between different receptors able to activate canonical and non-canonical NF-κB aids in the diversity of function for this signaling pathway. Much is known about this amazing pathway; much more is still to be known. In knowing this, we can address ways to enhance immune protection and vaccination as well as improve treatment protocols when NF-κB signaling gets out of control.

## Author contributions

MD: Headed the teamwriting and editing. ET: Contributed to writing and editing. CG: Contributed to writing and editing. All authors contributed to the article and approved the submitted version.
